# Effects of low-pass filtering on English speech-in-noise recognition in auditory-only and audiovisual modalities for late bilinguals and monolinguals

**DOI:** 10.1017/s1366728925100096

**Published:** 2025-06-13

**Authors:** Tiana Cowan, Emily Buss, Lori Leibold, Kaylah Lalonde

**Affiliations:** 1 Center for Hearing Research, Boys Town National Research Hospital, Omaha, NE, USA; 2 Department of Otolaryngology/Head and Neck Surgery, University of North Carolina, Chapel Hill, NC, USA

**Keywords:** bilingualism, audiovisual speech perception, speech-in-noise recognition, acoustic filtering, perception of speech produced by a talker with a face mask

## Abstract

The purpose of this study was to examine the impact of acoustic filtering and modality on speech-in-noise recognition for Spanish-English late bilinguals (who were exposed to English after their 5th birthday) and English monolinguals. All speech perception testing was conducted in English. Speech reception thresholds (SRTs) were estimated at 50% recognition accuracy in an open-set sentence recognition task in the presence of speech-shaped noise (SSN) in both low-pass and no-filter conditions. Consonant recognition was assessed in a closed-set identification task in SSN in four conditions: low-pass and no-filter stimuli presented in auditory-only (AO) and audiovisual (AV) modalities. Results indicated that monolinguals outperformed late bilinguals in all conditions. Late bilinguals and monolinguals were similarly impacted by acoustic filtering. Some data indicated that monolinguals may be more adept at integrating auditory and visual cues than late bilinguals. Theoretical and practical implications are discussed.

## 1. Introduction

Bilingual individuals who are exposed to their second language after their 5th birthday (late bilinguals) are more susceptible to interference from background noise when listening to speech in their second language compared to early bilinguals (Desjardins et al., 2019; Regalado et al., 2019; Mayo et al., 1997). The underlying mechanism accounting for group differences in susceptibility to noise is not yet fully understood. It has been posited that frequently encountered words have richer phonetic representations, which make them more robust to degradation from background noise (see Schmidtke, 2016). This hypothesis is supported by studies showing significant associations between speech-in-noise recognition and factors associated with language familiarity like age of exposure (Regalado et al., 2019; Shi, 2010, 2014) and language proficiency (Calandruccio & Zhou, 2014; Kilman et al., 2014; Rimikis et al., 2013). Poorer speech-in-noise recognition for bilinguals as compared to monolingual listeners suggests that bilinguals need more cues to support speech recognition. If that is the case, then bilingual listeners may also be more susceptible to the acoustic and visual speech cue degradation associated with speech produced when the talker is wearing a face mask. In this article, we examine how acoustic filtering impacts Spanish-English late bilinguals’ perception of English consonants and sentences in SSN and how visual cue availability supports consonant-in-noise recognition among late bilingual and monolingual groups.

### 1.1. Speech-in-noise recognition

Accurately perceiving speech in noise is challenging because of the overlap in acoustic energy between competing sounds (maskers) and the target signal (e.g., Brungart, 2001). In general, the greater spectrotemporal overlap between the target and masker predicts worse intelligibility. The target signal level relative to the noise level (SNR, signal-to-noise ratio) also impacts speech perception, with unfavorable SNRs making perception more challenging (Festen & Plomp, 1990; Fletcher & Galt, 1950). While features of the target speech like phonological variability, stress and prosody can affect intelligibility (Bregman, 1990; Buss et al., 2017), substantial within- and between-group differences may be accounted for by listener factors like age, cognitive function and linguistic knowledge as well (Buss et al., 2019; Ingvalson et al., 2017; and see also Rönnberg, 2003). One way to measure speech-in-noise abilities is to estimate the speech reception threshold (SRT), the minimum sound level, measured in decibels (dB), at which a listener can correctly recognize a predetermined proportion of target words. Another common way to assess speech-in-noise recognition is to measure the percent correct at one or more SNRs.

### 1.2. Linguistic factors related to bilingual speech-in-noise recognition

Late bilinguals tend to be more susceptible to noise when the target speech is in their second language compared to early bilinguals (e.g., Mayo et al., 1997), indicating that language history is an important predictor of speech recognition outcomes. For example, Shi (2012) assessed sentence-in-noise recognition at two SNRs, +6 and 0 dB, for 50 bilinguals with heterogeneous language backgrounds, most of whom learned English after 10 years of age (*n* = 42). Results indicated that later ages of exposure and ages of fluency predicted worse recognition of English sentences in noise (see also Shi, 2010). While language history is an important predictor of speech-in-noise recognition, linguistic dimensions of bilingual experience modulate its effect (Regalado et al., 2019; Shi, 2012, 2014, 2015; Shi & Farooq, 2012). For instance, target language proficiency and language dominance predict speech-in-noise recognition for bilinguals (Regalado et al., 2019; Shi, 2012). Regalado et al. (2019) found that late Mandarin-English bilinguals had higher (worse) English SRTs compared to early bilinguals and monolinguals and that language dominance scores assessed by the Bilingual Language Profile questionnaire (BLP, Birdsong et al., 2012) were negatively correlated with SRTs. Shi and Farooq (2012) evaluated degraded passage recognition in a sample of 36 bilingual adults to determine which linguistic and attitudinal factors predicted performance. Their results indicated that self-rated language proficiency accounted for the largest amount of variance among the tested factors (Shi & Farooq, 2012).

While speech perception is often assessed using sentence materials, consonant perception is also used. The demands of these tasks are different. In consonant perception tasks, listeners are typically presented with consonants in the context of single syllables without constraining semantic or syntactic context. Listeners identify consonants based on cues to voicing, manner of articulation and place of articulation. Across studies of consonant recognition, participants tend to confuse auditory consonants by place and voicing more often than manner (Benki, 2003). When listeners can see the talker’s face, the availability of place cues in the visual domain compensates for some common auditory confusions and the integration of auditory and visual cues can support more accurate speech perception compared to auditory cues alone (Grant et al., 1998; Grant & Seitz 1998; Braida, 1991).

Late bilinguals recognize consonants in their second language in quiet and in the presence of noise less accurately than monolinguals (Cutler et al., 2008; Garcia Lecumberri & Cooke, 2006). One reason for such differences is that the speech sound inventory of the first language seems to influence the second language inventory (Best, 1995; Navarra et al., 2005). For instance, the patterns of errors produced by bilinguals from different language backgrounds tend to differ, with errors predominantly reflecting the first language speech sound inventory of the listener (Cutler et al., 2004, 2008; Garcia Lecumberri & Cooke, 2006). Language history influences phonemic representations, such that speech sound categories tend to reflect properties of the language that the bilinguals had greater early exposure to (Pan et al., 2022), but may be updated based on the quantity, quality, and statistical distribution of input across both languages (Flege et al., 2021). Spanish and English have substantial overlap in consonant inventories; however, there are differences in phonetic features of consonants between languages that could impact late bilinguals’ perception of English consonants in the presence of noise.

Consonant recognition assesses phonetic feature processing, while sentence recognition additionally assesses more central language processing, like semantic and contextual processing (Mayo et al., 1997; Shi & Koenig, 2016). While sentence and consonant recognition tasks could rely to some extent on different auditory abilities and cues, few studies have assessed associations in performance across tasks. In examinations of monolingual speech perception, consonant and sentence recognition scores are correlated (Olsen et al., 1997; and see Grant et al., 1998 for evaluation in monolingual adults with hearing loss). Associations between performance on consonant and sentence recognition tasks could be different in bilingual listeners since linguistic dimensions that predict speech perception tend to be less correlated in late bilinguals compared to monolinguals (e.g., Luk & Bialystok, 2013). For instance, language proficiency and language use are less correlated in late bilinguals compared to monolinguals (Luk & Bialystok, 2013; Montrul, 2016; Schmeißer et al., 2016). Differences in these linguistic dimensions could impact phonetic feature processing and central language processing in distinct ways.

### 1.3. Considering the effects of acoustic filtering and visual occlusion on bilingual speech-in-noise recognition

At the start of the COVID-19 pandemic, face masks were widely mandated to reduce viral transmission (Centers for Disease and Control, 2023). While effective at mitigating viral spread, they also have consequences for communication. Face masks attenuate high-frequency speech information (Brown et al., 2021; Magee et al., 2021; Smiljanic et al., 2021) and remove or degrade access to visual speech cues, negatively impacting speech perception (Brown et al., 2021; Smiljanic et al., 2021; Toscano & Toscano, 2021). Considering the effect of listening to speech produced while the talker is wearing a face mask is important since the largest impact of low-pass filtering is observed in the absence of visual cues (Brown et al., 2021; Lalonde et al., 2022). For instance, Lalonde et al. (2022) tested English monolingual children and adults with typical hearing on consonant recognition, with and without visual cues and with a range of different low-pass filter cutoffs. Performance for all age groups was detrimentally impacted by the loss of visual cues and by low-pass filtering, with larger effects for more severe filtering; the largest effects were observed for place information. It is possible that degradation of acoustic and visual speech cues could yield a greater decrement in speech perception for bilingual adults, who tend to exhibit elevated susceptibility to background noise compared to monolinguals (e.g., Desjardins et al., 2019; Rogers et al., 2006).

Few studies have evaluated how bilingual adults perceive speech in AO and AV modalities, so there are few conclusions to draw from the existing literature. In general, both bilingual and monolingual adults recognize consonants more accurately in the AV modality compared to the AO one (Burfin et al., 2014; Kkese & Dimitriou, 2023). Studies comparing the magnitude of AV benefit between monolinguals and bilinguals have yielded mixed results. Xie et al. (2014) reported that bilinguals obtain *less* AV benefit during second language sentence-in-noise recognition compared to monolinguals and that target language proficiency modulates AV benefit for bilinguals. Findings from another study provided some indication that bilinguals may obtain *more* AV benefits in their second language compared to their first (Chauvin et al., 2022), suggesting that listeners might compensate for relatively limited linguistic experience by relying on visual cues in their second language. In the present study, we examine how late bilinguals and monolinguals integrate AV speech information in the presence of background noise to recognize English consonants in two acoustic filtering conditions.

### 1.4. The present study

The present study addressed the following research questions:

*Research Question 1: How does acoustic filtering impact sentence-in-noise recognition in late bilinguals and monolinguals?* Based upon prior research (e.g., Regalado et al., 2019), the prediction was that late bilinguals would exhibit higher SRTs (worse performance) for both low-pass filter and no-filter conditions compared to monolinguals.

*Research Question 2a*: *How does acoustic filtering impact consonant-in-noise recognition in late bilinguals and monolinguals?* Based on prior research in bilingual and monolingual groups, bilinguals were predicted to perform less accurately compared to monolinguals (e.g., Cutler et al., 2008). In both groups, consonant perception was expected to be worse as a consequence of acoustic filtering.

*Research Question 2b: How does modality impact consonant-in-noise recognition in late bilinguals and monolinguals?* Based on prior research, scores for both late bilinguals and monolinguals were predicted to improve in the AV modality compared to the AO one. An exploratory analysis examined the possibility that late bilinguals and monolinguals differed in their degree of benefit from visual cue availability when perceiving English consonants in noise (e.g., Chauvin et al., 2022; Xie et al., 2014).

*Research Question 3: How does English language proficiency predict both consonant and sentence recognition, and what is the relationship between English consonant recognition and sentence recognition in late bilinguals?* Based on prior work, English language proficiency was predicted to account for individual differences in SRTs. Exploratory analyses examined whether English language proficiency predicts consonant recognition in AO and AV modality conditions for late bilinguals, and whether bilingual consonant perception and sentence perception in the AO modality were correlated. Research Question 3 only assessed bilingual data since different monolingual participants completed consonant and sentence recognition tasks.

## 2. Methods and materials

The Institutional Review Board at Boys Town National Research Hospital approved all methods. Further, the authors assert that all procedures contributing to this work comply with the ethical standards of the relevant national and institutional committees on human experimentation and with the Helsinki Declaration of 1975, as revised in 2008.

### 2.1. Participants

Two groups of adults participated: late Spanish-English bilinguals and English monolinguals. Participants were 19–50 years of age and self-reported no history of language disability, learning disability, or neurological injury (e.g., traumatic brain injury). All participants passed a hearing screening, with air conduction thresholds ≤ 25 dB HL at octave frequencies from 0.25 to 8 kHz, bilaterally (American Speech-Language-Hearing Association, n.d.). Participants also passed a Snellen eye chart screening, indicating at least 20/30 vision bilaterally (corrected vision was screened as appropriate).

Additional inclusion criteria for late bilingual participants were self-report of exposure to Spanish from birth and English after 5 years of age and conversational fluency in both languages. Twenty late bilingual adults were recruited for the present study. One was excluded after failing a hearing screening. Nineteen late bilingual adults participated in the study (*M*_age_ = 38.7 years, male = 5, female = 14, other = 0). Their mean age of exposure to English was 11.9 years (*SD* = 5.1 range = 5–20 years).

The inclusion criteria for the monolingual participants were exposure to English from birth and only being conversationally fluent in English, by self-report. Two groups of monolingual English-speaking participants were recruited to complete each speech perception test. Ten English monolingual adult participants completed the sentence-in-noise recognition task (*M*_age_ = 30.6 years, male =6, female = 4, other = 0), and 10 English monolingual adults completed the consonant recognition task (*M*_age_ = 30.5 years, male =2, female = 8, other = 0). Four of the 10 monolingual participants completed both tasks. Data from 15 additional monolingual adults who participated in our previous study (Lalonde et al. 2022) are also presented for comparison to late bilingual participants (*M*_age_ = 22.3 years, male = 3, female = 12, other = 0).

### 2.2. Linguistic profile of bilingual participants

Language data were collected via the BLP (Birdsong et al., 2012) questionnaire to characterize the late bilingual participants. The BLP is a questionnaire where respondents self-report information about language history, proficiency, use and cultural attitudes and identity in four modules. The BLP questionnaire takes approximately 15 minutes to complete, and participants could elect to respond in either Spanish or English. Questions were read aloud to participants, if requested. The global language score is a weighted sum of scores from each subtest, with values ranging from 0 to 54.5; full results are reported in Appendix A.

Language dominance scores estimate the degree of language dominance from −218 to 218. In this study, BLP language dominance scores near 0 indicate “balanced” dominance across Spanish and English, negative values suggest Spanish dominance and positive values suggest English dominance. The mean language dominance composite score was −31.8 (*SD* = 53.3, range = −123.3–62.8), indicating bilingual participants tended to be Spanish dominant to some degree (Figure 1).

The Versant^™^ test of language proficiency directly assessed language proficiency in both Spanish (Spanish Versant^™^) and English (English Versant^™^). The Versant is a standardized test administered using web-based procedures. Scores on the Versant test of language proficiency range from 20 to 80, with these endpoints corresponding to “Basic” and “Proficient” users of the test language. Expressive and receptive language skills are assessed, including vocabulary, sentence comprehension and production, fluency and pronunciation, to yield composite proficiency scores. The Versant test of language proficiency is typically used in academic institutions, corporations and government agencies to evaluate language proficiency. It has also been used to estimate language proficiency in other studies of bilingual speech recognition under challenging listening conditions (Calandruccio & Buss, 2017; Cowan et al., 2023; Vicente et al., 2019).

The Versant tests of language proficiency were presented via laptop speakers, and participant responses were automatically scored. The order of the language of administration was randomized across participants.

For bilingual participants, Versant test of Spanish language proficiency scores (*M* = 75.5, *SD* = 5.3) were significantly higher than Versant tests of English language proficiency scores (*M* = 62.9, *SD* = 12.7), *t*(18) = 4.2, *p* < .001, with a mean difference of 12.6 points. Seventeen of the 19 bilingual participants had higher Versant scores in Spanish than in English. To review linguistic variables by participant, please refer to Appendix B.

### 2.3. Consonant recognition stimuli

The stimuli used to evaluate consonant recognition were previously used and described in Lalonde et al. (2022). In each trial, a female talker with a mainstream American dialect produced the carrier word, “Choose” followed by a target consonant-vowel syllable that combined one of 12 consonants with the vowel /i/: /bi/, /si/, /di/, /hi/, /ki/, /mi/, /ni/, /pi/, /∫i/, /ti/, /vi/ and /zi/. There were three unique recordings of each consonant-vowel syllable. Target stimuli were presented in SSN. The noise was gated on during each trial with 20-ms ramps at onset and offset. Participants were tested in a sound booth. Auditory stimuli were directly routed from a laptop to two loudspeakers (QSC, CP8; Costa Mesa, CA). Equipment was placed on a mat that was marked to ensure the correct configuration. Speakers were 23 cm from either side of the laptop, oriented toward the participants’ ears and positioned 40 cm from them. Participants used the trackpad to select the picture corresponding to the consonant-vowel syllable they just heard from a field of all 12 consonant options (e.g., a picture of a bumblebee corresponding to /bi/).

Test blocks included one presentation of each of the 36 stimulus recordings in a random order. For the present study, participants completed one test block in each of four conditions: AO no filter, AV no filter, AO low-pass filter and AV low-pass filter. The monolingual participants from Lalonde et al. (2022) completed testing in several other conditions, with two blocks per condition. Only the results from the first block of testing for the prior dataset were included in the current analyses, corresponding to the four conditions tested in the present study.

All late bilingual participants were tested at −8 dB SNR. Monolingual participants were tested at different SNRs to capture approximately equivalent performance between groups; those levels were −12 dB SNR (*n* = 5), −10 dB SNR (*n* = 5), −12 and −8 dB SNR (*n* = 5), −10 and 0 dB SNR (*n* = 5), or −8 and 0 dB SNR (*n* = 5). Some SNRs were tested after initial recruitment in an effort to match monolingual performance to that obtained for bilingual participants at −8 dB SNR. Therefore, not all monolingual participants completed all SNRs or tasks. In total, 10 monolingual participants were tested at each SNR. Condition and SNR order were randomized, with the exception that all testing at one SNR was completed before beginning testing at the next SNR.

Stimulus generation was described in Lalonde et al. (2022). Briefly, the low-pass filter condition simulates speech produced while wearing a Communicator^™^ mask, which is a single-use disposable mask with a transparent window. To simulate the acoustic filtering caused by the Communicator^™^ mask, the audio track of each stimulus recording was filtered using a 128-point FIR filter that was generated based on the difference between the long-term spectrum of speech recorded from a talker wearing no mask and speech recorded from that same talker wearing the Communicator^™^ mask. Acoustic filtering occurred mainly for frequencies above 2.5 kHz, with a mean attenuation of 7.8 dB between 2.5 and 10 kHz. To simulate the visual occlusion caused by the Communicator^™^ mask, a filled white polygon was superimposed over each frame of the videos based on the automatically determined position of points on the nose, mouth and jawline. To simulate the transparent window, there was a cutout in the mouth region of the polygon, providing access to visual speech information. This simulated Communicator^™^ mask occludes visual cues associated with the nose and jaw, but not lip cues. Detailed information about AV recording, initial processing and audio and AV face mask simulation can be found in Lalonde et al. (2022). The stimuli are available at https://osf.io/5wapg.

### 2.4. Sentence recognition stimuli

Sentence stimuli were lists 1 and 5 from the Basic English Lexicon (BEL; Calandruccio & Smiljanic, 2012) test. Each list of this corpus comprises 25 sentences that are simple in structure and have four keywords. An example sentence is, *The EGGS NEED MORE SALT*. BEL sentence lists are equated to rate of occurrence of keywords in the lexicon, the total number of affricates and fricatives and the number of syllables (Calandruccio & Smiljanic, 2012). These sentences were selected because they are regarded as linguistically appropriate for use with second-language learners (Rimikis et al., 2013). The same procedures used to filter and transform consonant materials were applied to the sentence stimuli. Participants heard sentence lists in either the no-filter or low-pass filter condition. Both the order and combination of list and acoustic filter conditions were randomized across participants, and participants never heard the same sentence more than once. Sentence recognition was assessed only in the AO modality.

### 2.5. Procedures for late bilingual participants

Participants first completed the consonant perception testing in a sound booth. A trained research assistant or research scientist sat with the participant in the booth for this task. Participants then completed open-set sentence recognition testing with stimuli presented diotically over headphones. The target stimuli were calibrated to 70 dB SPL at the position of the participant’s head, and the masker level was adjusted to achieve the desired SNR. Sentences were presented in noise at +3, −3, −6 and −9 dB SNR and in quiet. Each block of trials cycled through these stimulus conditions in order, from easiest (quiet) to hardest (−9 dB SNR), a total of 5 times. A new target was presented for each trial. Participants were presented a sentence and then asked to repeat what they heard. Their responses were scored for accuracy in real time by an experimenter seated outside the soundbooth using a custom script written in MATLAB (MathWorks, 2022). Participant performance was assessed by calculating the proportion of correct word recognition by SNR and acoustic filtering conditions.

### 2.6. Procedures for monolingual participants

Monolingual participants completed the consonant recognition testing and/or the sentence recognition testing. The four monolingual participants who completed both tasks completed them in different sessions, with sentence recognition testing in the first session and consonant recognition testing in the second.

### 2.7. Analysis

Scores by SNR were fitted using logit functions to estimate the psychometric function, and these functions were used to estimate the SRT associated with 50% correct (SRT_50_). For BEL sentence recognition, these functions spanned 0%–100%. For AO consonant recognition, that range was 8%–100% correct, to account for non-zero chance performance in a 12-alternative forced choice. For AV consonant recognition, the logit function spanned a range of 43%–100%; this value for the lower asymptote was selected to capture the performance supported by visual cues alone for this task (Lalonde et al. 2022). Psychometric functions were fitted using MATLAB (MathWorks, 2022).

Linear mixed models were used to evaluate logit-transformed proportion correct data. These models evaluated: how acoustic filtering impacts English sentence and consonant recognition in noise for late bilinguals and monolinguals; how modality impacts consonant-in-noise recognition across the two acoustic filtering conditions for late bilinguals and monolinguals; and how English language proficiency predicts sentence-in-noise recognition for late bilinguals. All models had random intercepts by the participant. Model assumptions were evaluated using standard procedures, including residual plot inspection, model fit assessment and review of variance component estimates. Linear mixed models were completed in R Studio software (R Studio Team, 2020) using the packages lme4 (Bates et al., 2015) and psych (Revelle, 2023).

## 3. Results

### 3.1. How does acoustic filtering impact sentence-in-noise recognition in late bilinguals and monolinguals?

Figure 2A,B shows psychometric function fits for monolingual and late bilingual sentence-in-noise recognition performance in the two acoustic filtering conditions. The mean performance at each SNR is shown with a circle. Overall, both groups were less accurate as the SNR decreased. The distribution of SRT_50_ values is plotted by condition in Figure 2D; gray lines connect points for individual participants. This figure shows that SNR has a similar effect on performance across groups and conditions. It also illustrates larger individual differences in performance for bilingual than monolingual participants, but similar effects of low-pass filtering for both groups. Figure 2C shows psychometric function fits for both monolingual and late bilingual groups for sentence-in-noise recognition performance in the two acoustic filtering conditions. On average, low-pass filtering elevated the SRT_50_ by 2.0 dB for bilinguals (−5.6 to −3.6 dB SNR) and by 2.4 dB for monolinguals (−7.8 to −5.4 dB). When monolingual participants’ data were assessed at the bilingual participants’ SRT_50_, they achieved a mean of 79.3% correct for the no-filter condition and 78.9% correct for the low-pass filter condition. In other words, monolinguals performed an average of approximately 29 percentage points better than bilingual participants at −5.6 dB SNR (no filter) and −3.6 dB SNR (low-pass filter).

Performance in quiet was estimated for both groups to characterize asymptotic performance. For monolingual participants, performance was at 100% in quiet for both the no-filter and low-pass filter conditions. For bilingual participants, mean performance in quiet -was 96.3% correct in the no-filter condition (range 85%–100%) and 98.2% correct in the low-pass filter condition (range 70%–100%). Performance was at 100% for 15/19 and 14/19 of late bilingual participants in these two conditions, respectively. These results indicate very good sentence recognition in quiet for both groups.

Linear mixed modeling assessed how acoustic filtering, SNR, group (i.e., language status) and the interaction between group and acoustic filtering predicted logit-transformed proportion correct data for the sentence-in-noise recognition task. Results are presented in Table 1. There was a significant main effect of group, signifying that monolinguals tended to perform better than late bilinguals. There was also a significant main effect of acoustic filtering, indicating that participants were more accurate in the no filter condition than in the low-pass filter condition. SNR predicted performance such that participants were less accurate as the SNR decreased. The group did not interact with the acoustic filtering condition, suggesting that late bilinguals and monolinguals were similarly impacted by low-pass filtering during sentence-in-noise recognition (Figure 2C).

### 3.2. How do acoustic filtering and modality impact consonant-in-noise recognition in late bilinguals and monolinguals?

Figure 3 shows monolingual and late bilingual consonant-in-noise recognition performance in the acoustic filtering and modality conditions. Panels A1–A4 show function fits to monolingual data as a function of SNR. Recall that the lower asymptotes for these functions were selected to represent performance in the absence of audible cues. Boxplots show the distribution of scores for bilingual participants, who were tested at −8 dB SNR. Panel B shows the distributions of scores for bilingual participants in the four conditions. Introducing the low-pass filter reduced performance by 6.0 points in the AV modality (74.3%–68.3%) and by 17.7 points in the AO modality (49.7%–32.0%). For comparison, fits data for monolinguals suggest that performance at −8 dB SNR fell by 10.4 points in the AV modality (93.3%–82.9%) and by 15.3 points in the AO one (68.0%–52.7%) with the introduction of the low-pass filter. Performance was also compared across groups by determining the SNR in fits to monolingual data corresponding to the performance of bilingual participants tested at −8 dB SNR. Those SNR values ranged from −11.6 to −10.5 dB for the four conditions. These SNRs were selected to be comparable to the approximately 2-dB difference in SRTs between groups observed for consonant-in-noise recognition in both acoustic filtering conditions.

Linear mixed models evaluated how language status predicted consonant-in-noise recognition when comparing late bilinguals tested at −8 dB SNR and monolinguals tested at either −10 dB or −12 dB SNR. Full results are presented in Table 2. These two levels were chosen because the mean performance for the bilingual participants tested at −8 dB SNR (56%) was similar to the mean performance of monolingual participants tested at −10 dB SNR (65%) and at −12 dB SNR (48%). In both models, the dependent measure was logit-transformed proportion correct performance by the participant. Independent variables were group, acoustic filter condition, and modality condition, and all interactions.

For the model with monolingual data obtained at −10 dB SNR, there were significant main effects of all three factors, consistent with the expectation of better performance for the monolingual group than the bilingual group, better performance for the no-filter than low-pass filter conditions and better performance for AV than AO conditions. None of the two-way interactions reached significance, but the three-way interaction of group, acoustic filter and modality was significant. The three-way interaction indicated that the combined effects of acoustic filter and AV benefit differ for the two groups. Reduced models for each group of participants confirmed that both bilinguals and monolinguals experienced significant benefits of having access to high-quality auditory (no filter) and visual information (AV modality); monolinguals experienced more than additive benefit from having access to both types of cues, whereas late bilinguals experienced less than additive benefit. The three-way interaction in the analysis comparing bilingual performance at the −8 dB level to monolingual performance at the −10 dB level could indicate that monolinguals are better at combining speech information across modalities (auditory and visual) than late bilinguals tested in their second language. This conclusion is tempered by the results of the analysis comparing performance for late bilinguals tested at −8 dB SNR and monolinguals tested at −12 dB SNR. This model replicated the significant main effects of acoustic filter and modality, and the nonsignificant two-way interactions, as reported above. In contrast to the analysis for monolingual data at −10 dB SNR, however, the three-way interaction for this model was not significant (*Coef* = 0.057, *t* = 1.36, *p* = 0.177).

### 3.3. How does English language proficiency predict both consonant-in-noise and sentence-in-noise recognition, and what is the relationship between English consonant and sentence recognition in late bilinguals?

Figure 4 shows the association between sentence-in-noise recognition, consonant-in-noise recognition and Versant test of English language proficiency scores for late bilinguals. Panels A1 and A2 show sentence scores at −5.5 dB SNR (derived from function fits) plotted as a function of consonant recognition scores at −8 dB SNR, both represented in percent correct. Versant test of English language proficiency scores are shown with a gradient color scale. Performance at −5.5 dB SNR was used so that performance would be comparable between tasks. Examination of Panels A1 and A2 shows that consonant-in-noise recognition is not correlated with Versant test of English language proficiency scores for either the no filter (*r* = .08, *p* = .743) or the low-pass filter condition (*r* = .06, *p* = .821), but sentence-in-noise recognition is significantly correlated with Versant scores in both the no filter (*r* = .60, *p* = .006) and low-pass filter (*r* = .60, *p* = .006) conditions. Examination of Panels A1 and A2 also indicates that consonant-in-noise recognition is not consistently correlated with sentence-in-noise recognition for either the no filter condition (*r* < .01, *p* = .999) or the low-pass filter condition (*r* = −.35, *p* = .138).

Linear mixed modeling assessed how English language proficiency predicted logit-transformed proportion correct data for the sentence-in-noise recognition task for late bilinguals. The independent variables were the main effect of SNR and the interaction between acoustic filtering and English language proficiency (Versant test of English language proficiency scores). The full results of this exploratory analysis are displayed in Table 3. The main effects of language proficiency and SNR were both positively associated with sentence-in-noise recognition accuracy (see Figure 4). There were no other significant effects. Versant scores predicted sentence-in-noise but not consonant-in-noise recognition (Table 4).

## 4. Discussion

The present study examined whether acoustic filtering and stimulus modality differentially impacted speech-in-noise recognition based on the language status and target language proficiency of listeners, given documented differences in susceptibility to noise between late bilinguals and monolinguals (e.g., Regalado et al., 2019; Rogers et al., 2006; Shi, 2015). As predicted, late bilinguals performed worse compared to monolinguals across conditions, indicating that late bilinguals listening to their second language recognize consonants and sentences in noise less accurately than monolinguals listening to speech with the same acoustic degradation. While late bilinguals exhibited worse performance compared to monolinguals in consonant and sentence recognition, both groups showed similar benefits from access to the full spectral bandwidth. In some conditions, monolinguals appeared to have greater benefit from access to the combination of full-bandwidth auditory cues and visual cues compared to late bilinguals, but this effect was not observed in all data. These results have practical implications for communicating in settings where talkers are likely to wear face masks, such as in hospitals.

### 4.1. Speech-in-noise recognition with degraded access to auditory or visual cues

Sentence recognition was assessed in the AO modality, and consonant recognition was assessed AO and AV, both across acoustic filtering conditions in bilingual and monolingual groups. English monolinguals were more accurate than late bilinguals at recognizing English consonants (in AO and AV modalities) and sentences (in AO modality) in the presence of noise. These findings are consistent with prior research on the effect of target language exposure on consonant perception (Cutler et al., 2004; Cutler et al., 2008; Garcia Lecumberri & Cooke, 2006) and with prior work showing group differences in speech-in-noise recognition between monolingual and late bilingual adults (Mayo et al., 1997; Regalado et al., 2019).

Looking across consonant and sentence recognition tasks, similar effects of low-pass filtering were found in both late bilingual and monolingual groups. In both AV and AO modalities, at SNRs resulting in more comparable consonant recognition accuracy, monolingual and bilingual participants were similarly impacted by the low-pass filtering. SRTs for sentence-in-noise recognition were also similarly impacted by the low-pass filtering. These findings could be interpreted to suggest that the same factor predicts speech recognition across adverse listening conditions for bilingual adults, particularly in the AO modality. Support for this hypothesis was also reported by Calandruccio et al. (2014), who showed that SRTs measured in steady-state noise predicted performance across three other modulated masker conditions.

For consonant-in-noise recognition, both groups exhibited substantial benefit from access to visual cues. Group comparisons were made at different SNRs to yield more comparable performance between groups than comparing both late bilinguals and monolinguals at −8 dB SNR. In the first analysis, comparing levels where English monolingual performance was just 9 percentage points more accurate than in late bilinguals, monolinguals showed more than additive benefit from having access to full-bandwidth auditory cues and visual cues, whereas bilinguals experienced less than additive benefit. Such a finding could point to monolinguals more efficiently integrating auditory and visual cues in the presence of masking compared to late bilinguals. However, in another model comparing performance at levels where late bilinguals were 8 percentage points more accurate than monolinguals, this interaction was not significant. Given the differences in model results at different comparison points, any observed group differences should be interpreted cautiously. Future studies may better characterize potential differences in visual cue benefit for consonant-in-noise recognition between monolinguals and late bilinguals by comparing AO and AV psychometric functions. This comparison is important because bilinguals and monolinguals may exhibit the greatest benefit at different points in the function (Xie et al., 2014).

Any differences in visual cue benefit for consonant-in-noise recognition between monolinguals and bilinguals may not generalize to sentence materials because there are differences in the processes underlying these tasks. Whereas consonant recognition assesses phonetic feature processing, sentence recognition additionally assesses more central language processing (Shi & Koenig, 2016). In monolinguals, visual cue benefit for consonant recognition only explains about 18% of the variance associated with individual differences in visual cue benefit for sentence recognition (Grant et al., 1998). Factors associated with language familiarity, such as age of exposure and language proficiency, may differentially impact visual cue benefits related to phonetic feature processing and central language processing. Future studies may characterize visual cue benefits across monolingual and late bilingual groups by assessing AO and AV psychometric functions for sentence recognition.

### 4.2. Differences in how English language proficiency predicts consonant and sentence recognition in noise for late bilinguals

For late bilinguals, English language proficiency, as estimated by the Versant test, accounted for individual differences in sentence recognition SRTs. Previous studies have shown that target language proficiency predicts sentence-in-noise recognition (e.g., Shi, 2012, 2013, 2015). However, English language proficiency did not account for individual differences in consonant-in-noise recognition across acoustic filtering conditions in the present study. It is possible that the association between English language proficiency and sentence-in-noise recognition points to listeners benefitting from the linguistic context in the BEL sentences and familiarity with target word forms, whereas listeners must rely upon sparse acoustic cues to accurately perceive consonants (Bradlow & Alexander, 2007; Shi & Koenig, 2016; Schmidtke, 2016). One future direction could be to expand upon this work by evaluating consonant recognition in bilinguals with different language profiles. For instance, since Spanish shares considerable overlap in consonant inventories with English, it is possible that different patterns would emerge if participants from language backgrounds with greater linguistic distance from the target language were tested. Nonetheless, this analysis serves as an important step in identifying linguistic factors that predict consonant-in-noise recognition in AV and AO modalities for late bilinguals.

### 4.3. The relationship between English sentence and consonant recognition in noise for bilinguals who are late learners of English

An assessment of the relationship between English consonant and sentence recognition in the AO modality in late bilinguals indicated no significant correlation. This could indicate that different linguistic factors predict bilingual consonant and sentence recognition. The timing and amount of target language exposure have been identified as the most influential factors predicting second language consonant perception (e.g., Flege & MacKay, 2004; Pan et al., 2022), whereas language proficiency is the most predictive linguistic factor for second language sentence recognition (e.g., Shi & Farooq, 2012). Procedural differences, such as the use of open set as compared to 12-alternate forced-choice task, the use of different target talkers and the measurement of accuracy vs. SRT also may have contributed to the lack of association.

### 4.4. Implications

At the start of the COVID-19 pandemic, face masks were commonly used to reduce viral spread, yet they had the unintended consequence of degrading the acoustic and visual speech cues available in many communication environments. Acoustic filtering in an everyday communicative situation motivated this examination of how late bilinguals perceive low-pass filtered speech in the presence of noise. Results suggest that both English monolinguals and late bilinguals experience greater difficulty recognizing speech in noisy environments when their communication partner is wearing a face mask, especially when that mask precludes visibility of the talker’s mouth and reduces high-frequency spectral content. Yet the practical impact of acoustic and visual cue degradation is greater for the late bilingual group, given their poorer performance overall. This suggests that late bilinguals require more favorable SNRs to achieve the same level of speech recognition accuracy as monolinguals regardless of whether their communication partner is wearing a face mask. In daily environments, like universities or hospitals, it may not be easy or possible to modify the SNR, which may result in late bilinguals being more susceptible to noise in these settings. Proactive steps should be taken to reduce barriers to successful communication, like offering translation services, providing quiet spaces and providing important information in writing in addition to verbally. These measures might be particularly beneficial for late bilinguals, who can require more speech cues to support accurate second-language speech recognition.

## Figures and Tables

**Figure 1.**
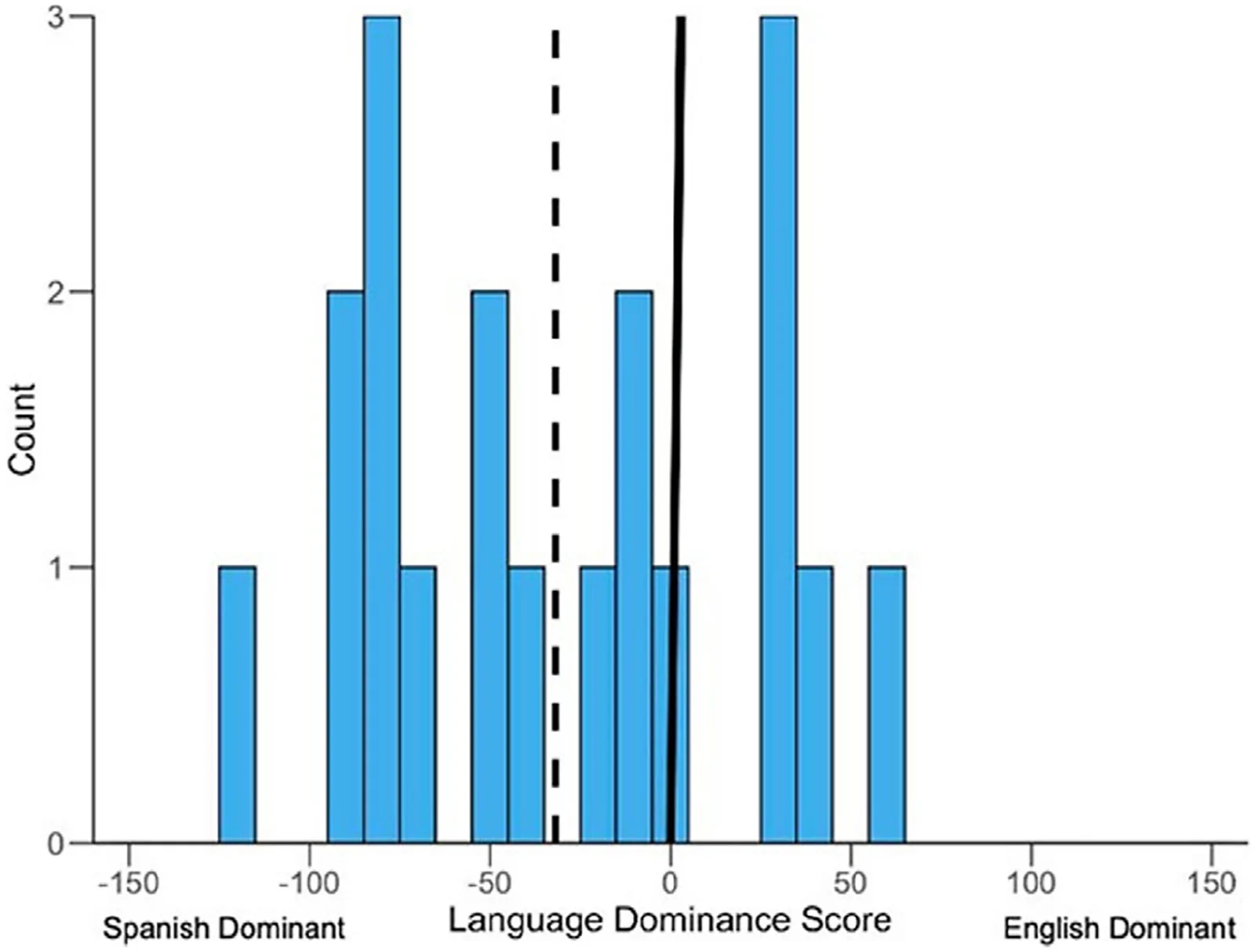
Histogram of language dominance scores from the bilingual language profile. This figure shows the distribution of language dominance scores from the Bilingual Language Profile (BLP). The full range of possible scores is −218 to 218, with scores in this study ranging from −123.3 to 62.8. The line at 0 corresponds to a “balanced” Language Dominance score. The dashed line shows the mean Language Dominance Score for the sample, −31.8. In general, most late bilingual participants self-reported some degree of Spanish language dominance. Five out of 19 participants had scores corresponding to English language dominance.

**Figure 2.**
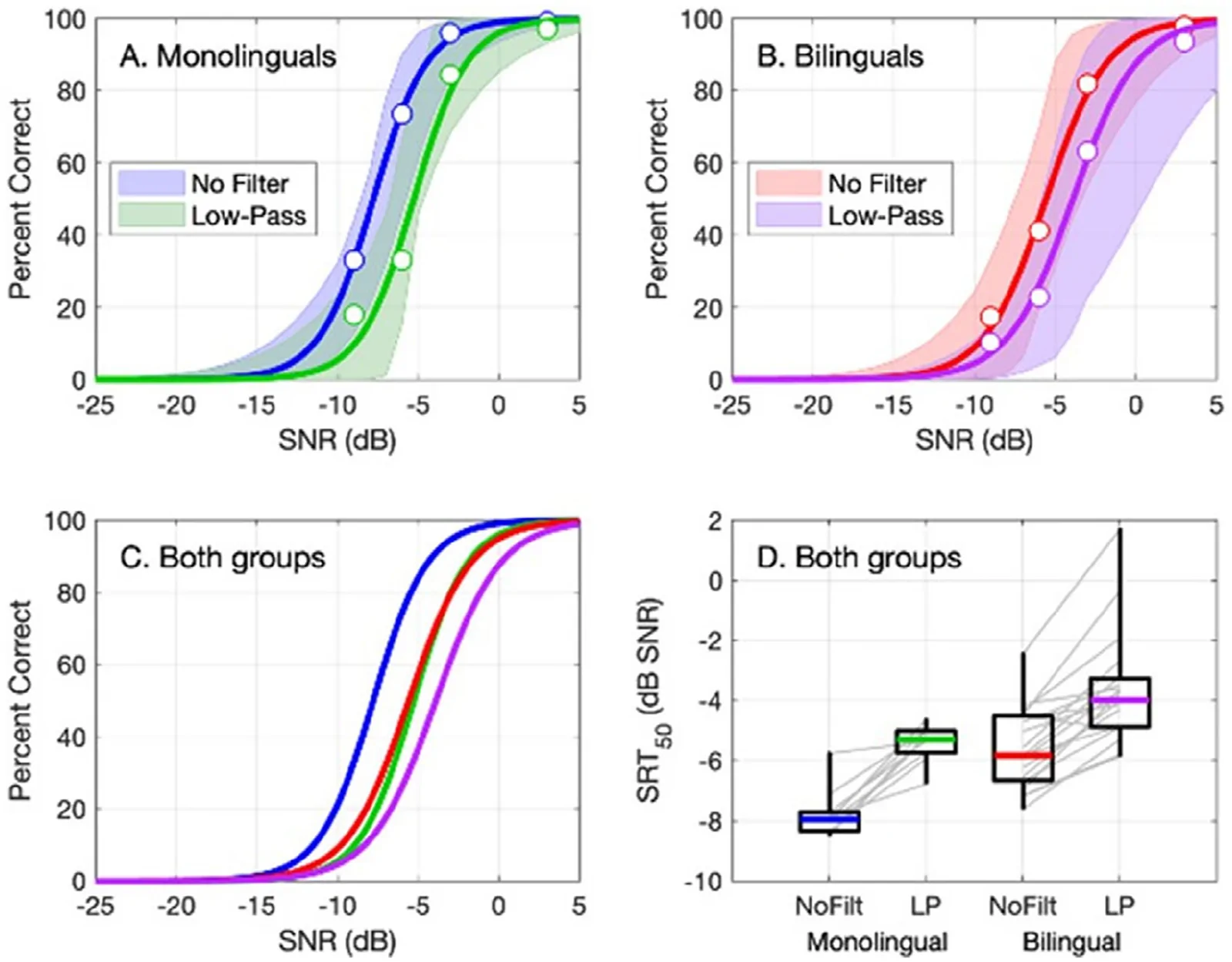
Results for the sentence-in-noise recognition task. Panels A and B show psychometric function fits for the monolinguals and bilinguals, respectively, plotted in percent correct as a function of SNR. The color indicates fits to low-pass (green and purple) and no filter (blue and red) data. Lines indicate fits to group data, and shaded regions indicate the 95% confidence interval by participant. The mean performance at each SNR is shown with circles. Panel C shows all four functions, following plotting conventions of panels A and B. Panel D shows the distribution of SRT_50_ values for each group and condition, as indicated on the x-axis; horizontal lines indicate the medians, boxes span the 25^th^ to 75^th^ percentiles and whiskers span the full range of values. Estimates for individual participants are connected with light gray lines.

**Figure 3.**
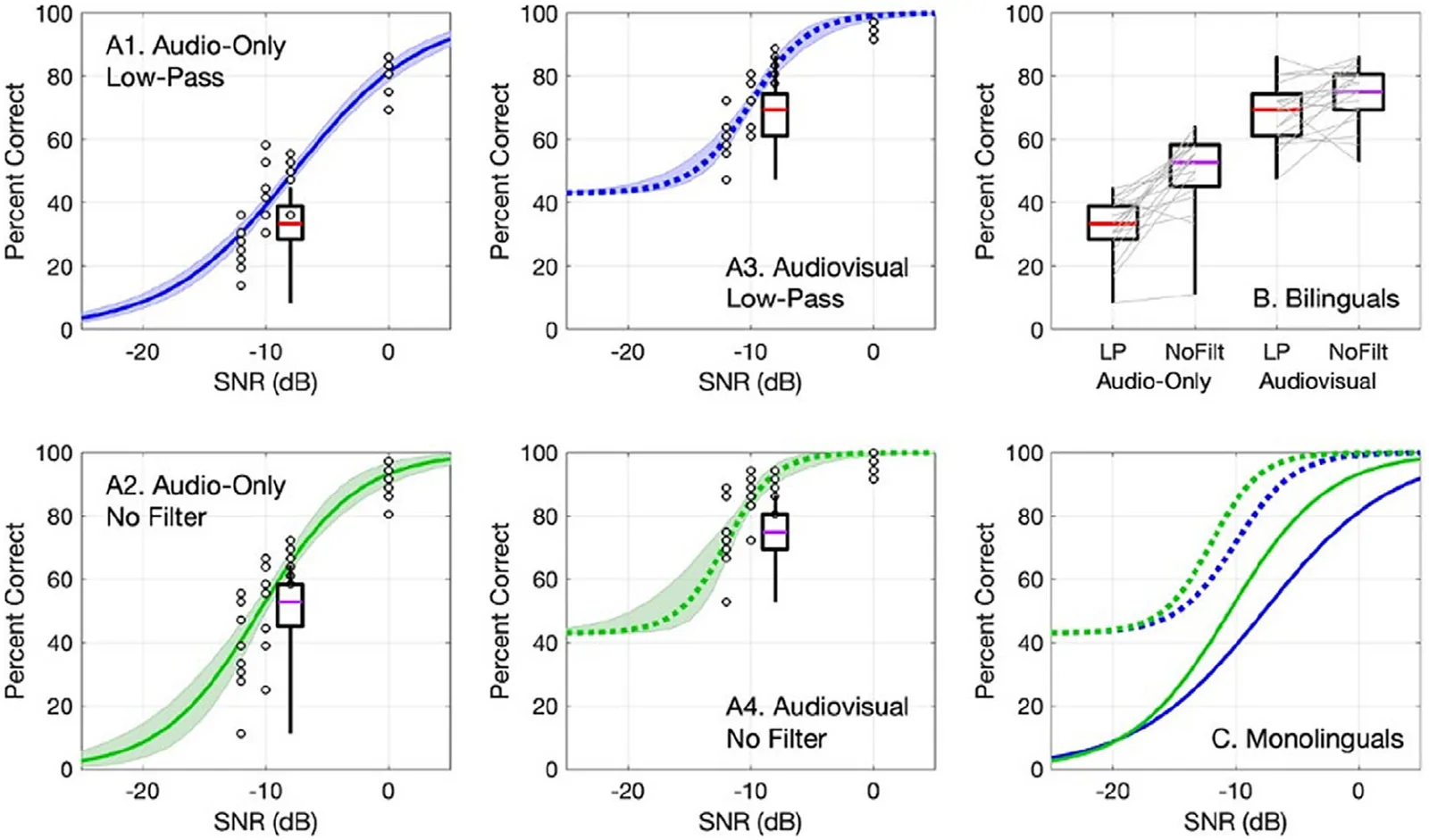
Percent correct consonant recognition as a function of SNR. Panels A1–A4 show results plotted separately by condition. Lines indicate psychometric functions fitted to monolingual data, indicated with open circles and shaded regions show the 95% confidence interval around this fit, computed with bootstrap resampling (n = 1000). Blue lines show performance in low-pass filter conditions, and green lines show performance in the no-filter condition. Boxes show the distribution of bilingual data obtained at −8 dB SNR in each condition; horizontal lines indicate the medians, boxes span the 25th to 75th percentiles, and whiskers span the full range of values. Panel B shows results for bilingual participants in all four conditions, indicated on the x-axis; light gray lines connect points for individual participants. Panel C shows functions fitted to monolingual data in all four conditions. Plotting conventions in panels B and C follow those in panels A1-A4.

**Figure 4.**
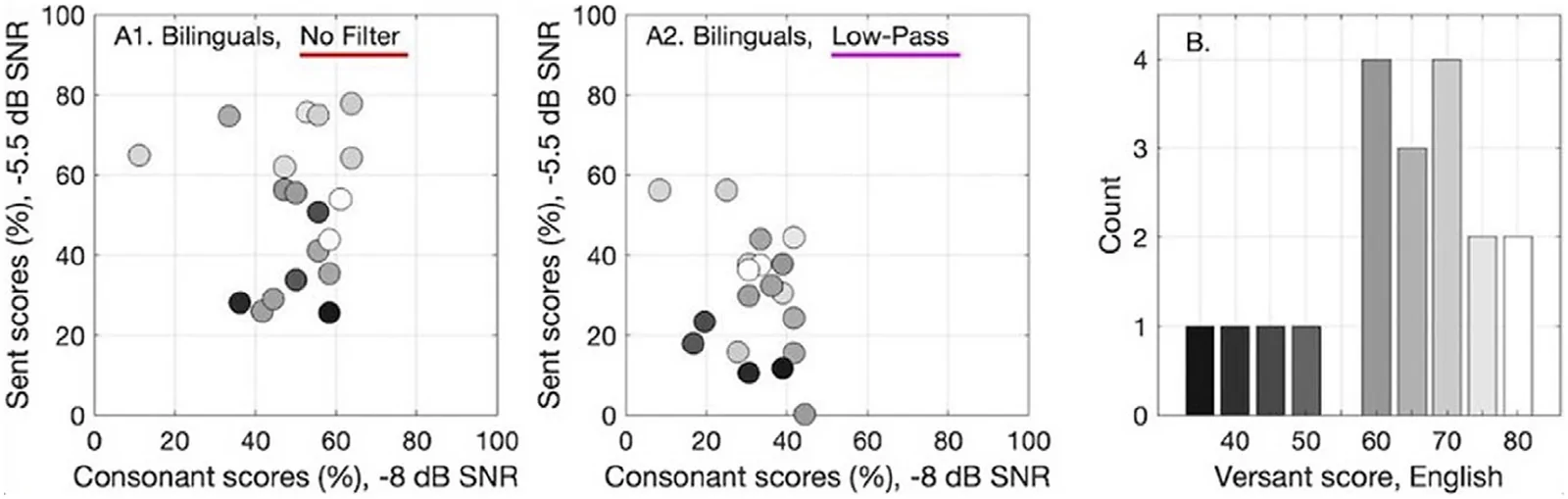
The association between sentence recognition, consonant recognition, and Versant scores for late bilinguals. Panels A1 and A2 show sentence scores at −5.5 dB SNR (derived from function fits) plotted as a function of consonant recognition scores at −8 dB SNR, both represented in percent correct. Panel A1 shows performance in the no-filter condition, and panel A2 shows performance in the low-pass condition. Color gradient from white to black indicates each participant’s score on the Versant English test of language proficiency, as defined in Panel B. Panel B is a histogram of Versant scores for the late bilingual participants.

**Table 1.** Linear mixed model results assessing the effect of acoustic filter (no filter or low-pass filter), group (monolingual or bilingual), and SNR on sentence-in-noise recognition

All participants	Coef.	*SE*	df	t-value	p-value
(Intercept)	.573	.027	70	21.05	<.001***
Group	−0.092	.032	61	−2.84	.01*
Acoustic filter	.145	.031	200	4.71	<.001***
SNR	.054	.002	200	26.66	<.001***
Acoustic filter: group	−.039	.038	200	−1.04	.301

*Note*: For this model, the dependent measure was logit-transformed proportion correct; the reference condition in the model was the late bilingual group, no filter condition, and 0 dB SNR. Significance codes .05 *, .01 **, and <.001 ***

**Table 2.** Linear mixed model results assessing the effects of acoustic filter (no filter or low-pass filter), group (monolingual or bilingual) and modality (AO or AV) on consonant-in-noise recognition

All participants	Coef.	*SEM*	df	t-value	p-value
Monolingual group at −10 dB SNR					
(Intercept)	0.511	0.070	27	7.32	< 0.001***
Group	0.243	0.070	27	3.48	0.002**
Acoustic filter	0.310	0.042	81	7.47	< 0.001***
Modality	0.750	0.042	81	18.06	< 0.001***
Group: mask	0.042	0.042	81	1.01	0.315
Group: modality	0.059	0.042	81	1.42	0.159
Filter: modality	0.014	0.042	81	0.35	0.730
Group: acoustic filter: modality	0.130	0.042	81	3.12	0.002**
Bilingual					
(Intercept)	0.268	0.088	18	3.05	0.007**
Acoustic filter	0.268	0.048	54	5.55	< 0.001***
Modality	0.691	0.048	54	14.28	< 0.001***
Acousticfilter: modality	−0.115	0.048	54	−2.38	0.021*
Monolingual					
(Intercept)	0.753	0.094	9	7.99	< 0.001***
Acoustic filter	0.352	0.068	27	5.16	< 0.001***
Modality	0.809	0.068	27	11.84	< 0.001***
Acoustic filter: modality	0.144	0.068	27	2.11	0.045*
All participants					
Monolingual group at −12 dB SNR					
(Intercept)	0.086	0.072	27	1.20	0.240
Group	−0.182	0.072	27	−2.53	0.018*
Acoustic filter	0.282	0.042	81	6.68	< 0.001***
Modality	0.712	0.042	81	16.89	< 0.001***
Group: mask	0.014	0.042	81	0.32	0.750
Group: modality	0.022	0.042	81	0.52	0.607
Acoustic filter: modality	−0.058	0.042	81	−1.37	0.174
Group: acoustic filter: modality	0.057	0.042	81	1.36	.177

*Note*: For these models, the dependent measures were logit-transformed proportion correct; the reference conditions in the models with three-way interactions between group, modality and acoustic filtering were the late bilingual group, no-filter condition and AO modality. Significance codes .05*, .01** and <.001***

**Table 3.** The effect of English language proficiency, acoustic filter (no filter or low-pass filter) and SNR on sentence-in-noise recognition for late bilinguals

Bilingual	Coef.	*SE*	df	t-value	p-value
(Intercept)	.144	.078	56	1.83	.071
Acoustic filter	.191	.108	130	1.77	.079
SNR	.058	.002	130	24.89	<.001***
Versant	.006	.001	55	4.56	<.001***
Acoustic filter: Versant	−.001	.002	130	−.81	.422

*Note:* For this model, the dependent measure was logit-transformed proportion correct; the reference condition was no filter. Significance codes .05*, .01** and <.001***

*Note:* Versant refers to the Versant English test of language proficiency score.

**Table 4.** The effect of English language proficiency, acoustic filtering (no filter or low-pass filter), modality (AO or AV) and SNR on consonant-in-noise recognition for late bilinguals

Bilingual	Coef.	*SE*	df	t-value	p-value
(Intercept)	.188	.094	30.66	2.00	.054
Acoustic filter	.053	.096	54	.55	.584
Modality	.242	.018	54	13.11	<.001 ***
Versant	.000	.001	30	.227	.822
Acoustic filter: Versant	.001	.002	54	.45	.651

*Note:* For this model, the dependent measure was logit-transformed proportion correct; the reference condition was no filter and AV conditions. Significance codes .05*, .01** and <.001***

*Note:* Versant refers to the Versant English test of language proficiency score.

## Highlights

- Monolinguals outperformed late bilinguals in all conditions
- Both groups’ recognition was similarly impacted by low-pass filtering
- Visual cue availability improved consonant recognition across groups

## Appendix A Bilingual Language Profile (BLP) scores by module and language for late bilingual participants (*n* = 19).

**Table:**

Module	Spanish scores Mean (SD) Range	English scores Mean (SD) Range
History***	44.4	27.7
	(8.3)	(12.4)
	29.1–54.5	7.3–46.8
Proficiency	49.47	46.72
	(6.49)	(6.61)
	36.32–54.50	31.80–54.5
Use	27.14	27.48
	(11.77)	(11.69)
	6.54–43.6	10.9–48.0
Cultural identity and attitudes**	49.46	33.80
	(7.96)	(13.63)
	31.8–54.5	11.4–54.5

Language dominance calculation			
	Spanish scores Mean (SD) Range	English scores Mean (SD) Range	Language dominance score Mean (SD) Range
Global language*	75.5	62.9	−31.8
	(5.3)	(12.7)	(54.3)
	66–80	36–80	−123.3–62.8

*Note:* Significance codes .05*, .01**, and <.001***.

## Appendix B Linguistic variables by participant.

**Table:**

Subject	AoE to English	BLP language dominance score	Versant English score	Versant Spanish score
1	6	−78.1	63	66
2	18	−92.2	63	80
3	10	26.0	70	80
4	5	42.5	75	66
5	20	−123.3	48	76
6	12	62.8	71	77
7	20	−94.7	39	80
8	13	−54.0	73	80
9	10	−19.9	62	75
10	9	−14.7	60	72
11	9	−7.8	71	77
12	5	35.0	72	67
13	20	−52.7	46	79
14	12	−37.3	36	67
15	20	−71.6	64	79
16	10	−75.9	79	80
17	7	4.2	61	74
18	12	−84.1	62	80
19	8	32.0	80	80

*Note*: AoE refers to age of exposure in years.
